# Integrated fertilization with brazide-mediated regulation improves stem mechanical strength, yield, and water-use efficiency of blueberry under semi-arid conditions

**DOI:** 10.3389/fpls.2026.1917640

**Published:** 2026-09-28

**Authors:** Xinchao Ma, Hailiang Chen, Xuefeng Zheng, Yongming Wang, Yang Bai, Xuefei Guo

**Affiliations:** 1 Xinjiang Production & Construction Corps Key Laboratory of Facility Agriculture, Tarim University, Alar, China; 2 Yunnan Wangyueberry Agricultural Technology Co., Ltd., Yunnan, China; 3 1st Agricultural Division, Xinjiang Production and Construction Corps, Agricultural Science Research Institute, Xinjiang, China; 4 Chaoyang County Agricultural Machinery Technology Extension Service Center, Liaoning, Liaoning, China

**Keywords:** blueberry production, economic benefit return, integrated fertilization, lodging stress, nitrogen remobilization and uptake, plant growth regulator, roots development

## Abstract

Water scarcity and the potential for soil degradation and environmental problems due to excessive fertilizer use are major challenges to achieving green, sustainable blueberry production with high water-use efficiency. Plant growth regulators (PGRs) play a crucial role in improving blueberry production. This study investigated the effects of CFB1: chemical fertilizer with 1 µmol l^-1^ brazide; CFB2: chemical fertilizer with 3 µmol l^-1^ brazide; CFB3: chemical fertilizer with 5 µmol l^-1^ brazide; OFB1: organic fertilizer with 1 µmol l^-1^ brazide; OFB2: organic fertilizer with 3 µmol l^-1^ brazide; OFB3: organic fertilizer with 5 µmol l^-1^ brazide; BFB1: biofertilizer with 1 µmol l^-1^ brazide; BFB2: biofertilizer with 3 µmol l^-1^ brazide; BFB3: biofertilizer with 5 µmol l^-1^ brazide; CK: no fertilizer with water foliar spray on blueberry resistance to water and lodging stress, N uptake and remobilization, root growth, resource use efficiencies, and economic benefits during the 2022 and 2023 study periods. Our results showed that under the OFB2 treatment, the blueberry stalk lodging index and lodging rate decreased by 25.8% and 31.5%, respectively, through changes in the morphological structure and enhanced soil respiration and water use efficiency (WUE), which increased blueberry yield and substantially reduced crop evapotranspiration (ETc) and greenhouse gas emissions. Under organic fertilizer combined with 3 µmol l^-1^ brazide application, leaf area index (LAI), Pn, root growth, and bleeding sap were observed to have improved at various growth stages of blueberry. Further, maximum water use efficiency (58.2%, WUE), nitrogen use efficiency (39.2%, NUE), nitrogen recovery efficiency (34.8%, NRE), and nitrogen partial productivity (64.8%, NPP) were obtained under the OFB2 treatment, compared with the CK treatment. In addition, the OFB2 treatment increased N uptake and the percentage of total N content, finally improving the number of flower buds (30.8%), number of new shoots (49.0%), blueberry yield (68.9%), and net profit, respectively. Overall, 3 µmol l^-1^ brazide with organic fertilizer exhibited the highest blueberry production, antioxidant defense system, NUE, and economic benefits, maintaining high WUE, and could be a promising approach to the sustainable development of blueberry production performance under semi-arid field conditions.

## 1 Introduction

The use of organic fertilizers is considered a sustainable agricultural practice because it improves the soil’s chemical, physical, and biological properties (Liu et al., 2021). However, due to the slow nutrient release nature of organic fertilizers, the organic nutrients contained must be mineralized into inorganic forms for crops to absorb and utilize them (Chen et al., 2011; Jayasinghege et al., 2024). Reducing the use of chemical fertilizers and using a combination of organic and inorganic fertilizers are gradually recognized as a direction for sustainable agricultural development (Zhai et al., 2023). As a result, blueberry yield and resource utilization efficiency are significantly improved (Cheng et al., 2023). Furthermore, biofertilizers and organic fertilizers are also considered alternatives to chemical fertilizers, and their main components are beneficial microorganisms associated with the roots (Peng et al., 2014; Bañados et al., 2006). Plant growth-promoting rhizosphere bacteria (PGPR) are important microbial inoculants and are widely used as biofertilizers because they promote crop growth (Quemada and Gabriel, 2016; Naservafaei et al., 2021). Brassinolide (BR), a phytohormone belonging to the brassinosteroids family, is involved in different plant physiological processes, including adaptation to various abiotic and biotic stresses (Naservafaei et al., 2021). Bacillus, an important plant growth-promoting rhizosphere bacterium, promotes plant development and enhances plant resistance to various ecological stresses (Ali and Khan, 2021). Due to its environmental friendliness, Bacillus has been approved as a commercial biofertilizer, which can be used alone or in combination with organic fertilizers (Li et al., 2021). BR is an effective, environmentally friendly, and safe natural plant growth regulator that promotes plant growth (Fariduddin et al., 2009; Naservafaei et al., 2021). BR thoroughly enhances stress resistance by enhancing antioxidant defenses and photosynthetic capacity in plants (Leskovar and Othman, 2018). BR protects the photosynthetic mechanism under water stress, improves chlorophyll stability, and increases leaf area index, thereby promoting photosynthesis (Naservafaei et al., 2021; Wu et al., 2023). However, BR is low in content and requires complex synthetic processes, leading to high production costs. Therefore, the development of alternative analogs is essential. One such analogue is brazide, which has enhanced activity and has shown great potential for improving plant growth and development in blueberry (El-Mashad and Mohamed, 2012; Lv et al., 2020). The efficiency of brazide in blueberry under various levels of water stress remains unclear, however.

Declining soil fertility and water scarcity are two major factors that reduce blueberry productivity under semi-arid field conditions (Godfray et al., 2010; Ali et al., 2019). The threat to crop production due to water scarcity ultimately results in annual income losses of US$6.4 billion worldwide (Hao et al., 2015; Bañados et al., 2012). In China, approximately 60% of cultivated land is dry-land, and approximately 40% is affected by semi-arid field conditions, of which 40% face water scarcity problems (China Agricultural Yearbook Editorial Committee, 2014). Therefore, water scarcity is a significant factor limiting blueberry productivity in dry-land regions (Yu et al., 2022). Lodging stress is a complex phenomenon influenced by multiple factors, including excessive planting density, excessive nitrogen fertilizer application, heavy rain, strong winds, hail, topography, soil type, pests, and diseases (Zhang et al., 2022). Lodging stress is caused by stem breakage or by root lodging (Yao et al., 2023). Lodging stress reduces canopy photosynthesis, negatively impacting blueberry yield (Xu et al., 2019; Strik, 2016). In blueberry, stems play a critical role in stem lodging resistance (Guo et al., 2022). Thus, the integrated use of nitrogen fertilizer can maximize the use of agro-ecosystem resources, decrease the risk of pollution, and ensure food safety.

Lodging is one of the major limiting factors affecting the nutritional quality and production of conventional cereals worldwide (Sposaro et al., 2008; Chen et al., 2011). Lodging disrupts the plant canopy structure, reduces nutrient uptake, reuse, and utilization efficiency, and, coupled with poor light penetration, ultimately leads to decreased food quality and blueberry yield (Wang et al., 2015). Furthermore, in addition to yield reduction, soil residue also decreases the efficiency of mechanical harvesting, which increases harvesting time and labor costs (Kuai et al., 2015). Therefore, a novel strategy to improve the lodging tolerance of crops is to enhance the physical strength of blueberry stems (Wang et al., 2021; Ehret et al., 2015). Lignin, the main component of secondary cell walls, provides mechanical strength and reduces stress caused by lodging in plants (Wang et al., 2015; Liu et al., 2021). The greater the lignin accumulation in the blueberry stem, the stronger the lodging resistance (Ehret et al., 2014; Weng et al., 2017). Higher physical strength and cellulose content in basal nodes can reduce lodging rates (Peng et al., 2014). Although nitrogen fertilizer use has increased significantly to increase yields, nitrogen use efficiency has not increased correspondingly (Zhang et al., 2018; Bryla et al., 2012). Current production management practices, particularly the unscientific use of growth regulators and fertilizers, have resulted in significant resource waste, increased plant failure, and reduced crop production (Paltineanu et al., 2018; Yang et al., 2019). To address these issues, it is important to develop scientific fertilization and PGR strategies to improve blueberry yield, nitrogen use efficiency (NUE), and water use efficiency (WUE) and decrease evapotranspiration (ET), greenhouse gas (GHG) emissions, and the risk of crop failure.

Earlier research work has reported that the combined use of biofertilizer, chemical fertilizers, and organic fertilizers is an ecologically friendly and sustainable agricultural practice that significantly increases blueberry yield and improves soil nutrients and microbial ecosystems (Behnamnia, 2015; Chen et al., 2021). But the direct mechanisms by which plant growth regulators, when combined with other fertilizers, affect blueberry growth remain unclear. Furthermore, studies on the effects of different concentrations of the plant growth regulator brazide combined with fertilization performance under semi-arid conditions on blueberry production, WUE, and NUE are rarely reported. Such studies are crucial for maximizing agricultural and economic benefits while minimizing environmental impacts. This study hypothesized that the combined application of biofertilizer and chemical and organic fertilizers using the plant growth regulator brazide would promote blueberry growth and improve WUE, NUE, and economic return. The following objectives were set: (1) to determine the effects of the brazide plant growth regulator with integrated fertilization management on blueberry growth, production, NUE, WUE, and economic return; (2) to evaluate the effects of brazide concentrations on blueberry growth, yield, and water productivity under integrated fertilization management performance under semi-arid field conditions and to assess the potential of brazide to improve NUE; and (3) to determine which among the tested treatments of brazide concentration and fertilization method can offer the best performance and simultaneously optimize blueberry yield, WUE, photosynthesis, NUE, and economic return. This study provides a theoretical basis for integrating and applying various concentrations of brazide to synergistically increase resource utilization efficiency and blueberry production.

## 2 Materials and methods

### 2.1 Site description and research design

Field trails were conducted in 2022 and 2023 at the research station in Xinjiang, China, with latitude (44°24’09” N), and longitude (86°09’17” E) of the experimental site. The soil at the experimental station was clay loam, and its physicochemical properties are shown in **Table 1**. Compared with inland areas at the same latitude, this experimental station has a typical warm temperate monsoon climate, with more precipitation and a milder climate than inland areas. The annual mean temperature is 16.81 °C; the annual precipitation is 468 mm; and the annual sunshine hours are 1,195 hours.

**Table 1:** The chemical properties of the experimental site of the soil layers (0–20 cm).

Year	pH	SOM (g kg^-1^)	TN (g kg^-1^)	TP (g kg^-1^)	TK (g kg^-1^)	AP (mg kg^-1^)	AK (mg kg^-1^)
2022	8.18	14.87	1.13	1.05	17.19	21.02	160.18
2023	8.11	14.33	1.10	1.04	17.22	20.91	161.12

SOM, soil organic matter; TN, total nitrogen; TP, total phosphorus; TK, total potassium; AP, available phosphorus; AK, available potassium.

This study was conducted under a randomized complete block design (RCBD) with a single-factor experiment and three replications. The area of ​​each experimental plot was 240 m² (8 m × 30 m) in size. In the field study, CFB1: chemical fertilizer with 1 µmol l^-1^ brazide; CFB2: chemical fertilizer with 3 µmol l^-1^ brazide; CFB3: chemical fertilizer with 5 µmol l^-1^ brazide; OFB1: organic fertilizer with 1 µmol l^-1^ brazide; OFB2: organic fertilizer with 3 µmol l^-1^ brazide; OFB3: organic fertilizer with 5 µmol l^-1^ brazide; BFB1: biofertilizer with 1 µmol l^-1^ brazide; BFB2: biofertilizer with 3 µmol l^-1^ brazide; BFB3: biofertilizer with 5 µmol l^-1^ brazide; CK: no fertilizer with water foliar spray (**Table 1A**). The plant growth regulators were sprayed at a rate of 450 L ha^-1^ using a 5 L precision sprayer at the full bloom stage (FBS) and early green fruit stage (EGFS). Inorganic fertilizers used in this study included urea (46% N), single superphosphate SSP (18% P_2_O_5_), and muriate of potash (60% K_2_O) as sources of nitrogen, phosphorus, and potassium. Organic fertilizers were produced using sheep manure and contained 29 (g kg^-1^) OM, 1.2 (g kg^-1^) total nitrogen, 0.9 (g kg^-1^) total phosphorus, and 0.6 (g kg^-1^) total potassium. Biofertilizer contained more than 2 × 10^9^ CFU g^-1^ bacteria of *Bacillus subtilis* and *Bacillus amyloliquefaciens* and used starch as the main medium. A total fertilizer dose of 270 kg ha^-1^ nitrogen, 105 kg ha^-1^ phosphorus, and 90 kg ha^-1^ potassium was applied. The blueberry variety ‘Britewell’ was used as the experimental material, which is 6 years old. The berries were collected during the same ripening stage in both study years. Planting was carried out on an elevated bed with a distance of 1.5 m between plants. We maintained a 1-meter-wide space to prevent rainwater and nitrogen from leaking between adjacent plots. In both growing seasons, there was no irrigation, and weeds were artificially controlled.

**Table 1A:** The treatments details.

Treatments	Full abbreviations
CFB1	Chemical fertilizer with 1 µmol l^-1^ brazide
CFB2	Chemical fertilizer with 3 µmol l^-1^ brazide;
CFB3	Chemical fertilizer with 5 µmol l^-1^ brazide
OFB1	Organic fertilizer with 1 µmol l^-1^ brazide
OFB2	Organic fertilizer with 3 µmol l^-1^ brazide
OFB3	Organic fertilizer with 5 µmol l^-1^ brazide
BFB1	Biofertilizer with 1 µmol l^-1^ brazide
BFB2	Biofertilizer with 3 µmol l^-1^ brazide
BFB3	Biofertilizer with 5 µmol l^-1^ brazide
CK	No fertilizer with water foliar spray

### 2.2 Photosynthesis rate and leaf area

The green leaf area (LA, cm^2^) per plant was measured as follows by Equation 1, 2:

(1) LA=L×W×0.75

(2) LAI=LA/GA

where LA (cm^2^) is the green leaf area and GA (cm^2^) is the ground area.

Five marked leaves were selected from each plot, and their photosynthetic rates were measured using a Li-6400 portable photosynthesis system between 9:00 and 11:00 AM on sunny days at various growth stages. The CO_2_ concentration in the leaf chamber was set at 380 μmol mol^−1^ and the photosynthetically active radiation was set at 1100 μmol m^-2^ s^−1^.

The GHGI (kgCO_2_-eq kg^-1^ yield) was calculated by Equation 3 using net GWP per blueberry yield (Ali et al., 2017):

(3) GHGI = NetGWP/Yb 

### 2.3 Lodging rate

The stem lodging resistance index (%) was calculated according to Zhang et al. (2015).

Stem lodging resistance index = stem bending resistance strength/center of gravity height; observation of lodging in plants included stem lodging and root lodging.

The lodging rate in each plot was calculated using the following formula (Equation 4):

(4) Lodging rate (%)=number of lodged plants/total number of plants×100

Lodging rates were expressed as a percentage of the number of plants lodged to the total number of plants in each plot.

### 2.4 Nitrogen uptake and use efficiencies

The total plant N concentration was measured using the Micro Kjeldahl method.

Nitrogen use efficiency (NUE), nitrogen recovery efficiency (NRE), and nitrogen partial factor productivity (NPFP) were calculated using the following formulas (Equations 5–7):

(5) $$\text{NUE }({\text{kg kg}}^{-1})=(\text{YN}-\text{Y}0)/\text{N}$$

(6) NRE (%)=(UN−U0)/N×100%

(7) $$\text{NPFP }({\text{kg kg}}^{-1})=\text{YN}/\text{N}$$

where Y_N_ and Y_0_ are the yields (kg ha^-1^) in the nitrogen-applied and non-nitrogen-applied plots, U_N_ and U_0_ are nitrogen uptake (kg ha^-1^) in the nitrogen-applied and non-nitrogen-applied plots, and N is the amount of nitrogen applied (kg ha^-1^).

### 2.5 Leaf physiological activities

Six fresh blueberry leaves were collected to evaluate leaf physiological activity. Chlorophyll content was determined after extraction with 80% acetone following the procedures and equations described by Lichtenthaler and Wellburn, (1983). Soluble protein content was measured using the Coomassie Brilliant Blue G-250 method. Soluble sugar concentration was determined using the anthrone colorimetric method, whereas superoxide dismutase (SOD) activity was assessed using the nitroblue tetrazolium photochemical reduction method. Chlorophyll, SOD activity, and malondialdehyde (MDA) contents were analyzed according to the methods reported by Strik and Vance, (2017) and Dehghan et al. (2020). The growth rate of new shoots was recorded in May by randomly selecting current-year shoots and monitoring their development. Flower bud differentiation was primarily assessed in January, during which the total number of buds was also determined. The crushing strength (CS) was determined using a stem strength tester equipped with a 1 cm² probe (Shijiazhuang Shiya Technology Co., Ltd.); the researchers applied pressure vertically at a constant speed. The hemicellulose, lignin, and cellulose contents in the sieved samples were determined using the Van Soest method (Van Soest et al., 1991).

### 2.6 Resource use efficiencies and economic return

Soil respiration was measured using an LI-8100 automated soil CO_2_ flux system manufactured by LI-COR Biosciences, Lincoln, Nebraska, USA, equipped with integrated temperature and humidity sensors. Seasonal crop evapotranspiration (ETc, mm) was estimated using the equation described by (Enrique, 2023). According to Yang et al. (2002), the root bleeding sap collection method is most suitable for the collection of maize root sap. According to the study of Ali et al. (2019), the transport rate was expressed as ml h^-1^ root^-1^.

The ET rate was calculated as follows (Equation 8):

(8) $$\text{ET}=\text{P}+\text{I}+\text{C}+({\text{SW}}_{1}-{\text{SW}}_{2})-\text{D}-\text{R}$$

where P is the rainfall; I is the irrigation; C is the upward flow into the root zone; SW1 is the soil water storage at planting time, SW2 is the soil water storage at maturity stage, and both SW1 and SW2; D is the downward drainage; and R is the surface runoff. The groundwater table remained at a depth of 80 m, so the upward flow into the root was negligible. The trial field was flat, so runoff was never observed in the experiment.

The water use efficiency (WUE) was determined using Equation 9 (Huang et al., 2005; Payero et al., 2008):

(9) WUE=Y/ET

where Y (kg ha^−1^) is the blueberry yield; ET is the crop evapotranspiration.

The economic return of blueberry was calculated as follows (Equation 10).

(10) NI=TI−(FE+VE)

In the equation, NI represents net income (CNY ha^-1^), TI denotes total income (CNY ha^-1^), (FE) refers to fixed expenditures (CNY ha^-1^), which include irrigation, herbicide application, machinery operation, and labor costs, whereas (VE) indicates variable expenditures (CNY ha^-1^), primarily associated with fertilizer inputs.

### 2.7 Statistical analysis

The three replicates of data for each treatment were analyzed, and significant differences between treatments were determined based on the least significant difference (LSD) test. For all parameters, a single-factor analysis of variance (ANOVA) was conducted using the SPSS 13.0 program (SPSS Inc., Chicago, IL, USA). Differences were considered significant when *P* < 0.05. The means among treatments were compared based on the least significant difference (LSD) test. All the analyses were performed with a confidence level of 95% using SPSS 13.0 (SPSS Inc., Chicago, IL, USA), and data obtained from each sampling event (growth stage) were analyzed separately. The year data were analyzed separately. Data analyses and all figures were prepared using Excel 2010, SPSS 13.0, Origin 2019, and Sigma Plot 12.5. The ANOVA assumptions were met without any transformations.

## 3 Results

### 3.1 Leaf area index and N remobilization, uptake use efficiencies

As shown in **Figure 1**, foliar application of the plant growth regulator brazide combined with fertilization affected the leaf area index (LAI) in both study years. LAI generally increased with increasing concentration of brazide foliar application, up to a concentration of 3 µmol L^-1^. During the growth stages of early green fruit state (EGFS), late green fruit stage (LGFS), and fruit coloring state (FCS), the LAI of the B1 and B3 treatments was lower than that of the B2 treatment. During the LGFS stage, the average LAI of treatments the B3 and B1 treatments was lower than that of the OFB2 treatment in both study years. Different concentrations of the plant growth regulator brazide combined with fertilization affected LAI. Under the OF treatment, blueberry plant given B2 treatment had the highest LAI during the EGFS stage, higher than those under other combined fertilization treatments; however, during the EGFS, LGFS, and FCS growth stages, the LAI values ​​of the OFB2 and BFB2 treatments were higher than those of all other treatments. Under the B2 treatment, the foliar spraying of brazide (a plant growth regulator) combined with organic fertilizer resulted in considerably greater LAI at all growth stages of blueberry compared with the chemical fertilizer and biofertilizer treatments. However, non-significant variances were observed among the treatments at the LPBS stage. At the LPBS and FBS growth stages, there were non-significant variances among the fertilization treatments under different concentrations of brazide foliar spraying, while the CK treatment showed significantly lower LAI at all growth stages compared with other fertilization treatments. The trends in blueberry LAI in 2022 and 2023 were consistent, with minimal differences between the 2 years.

**Figure 1:**
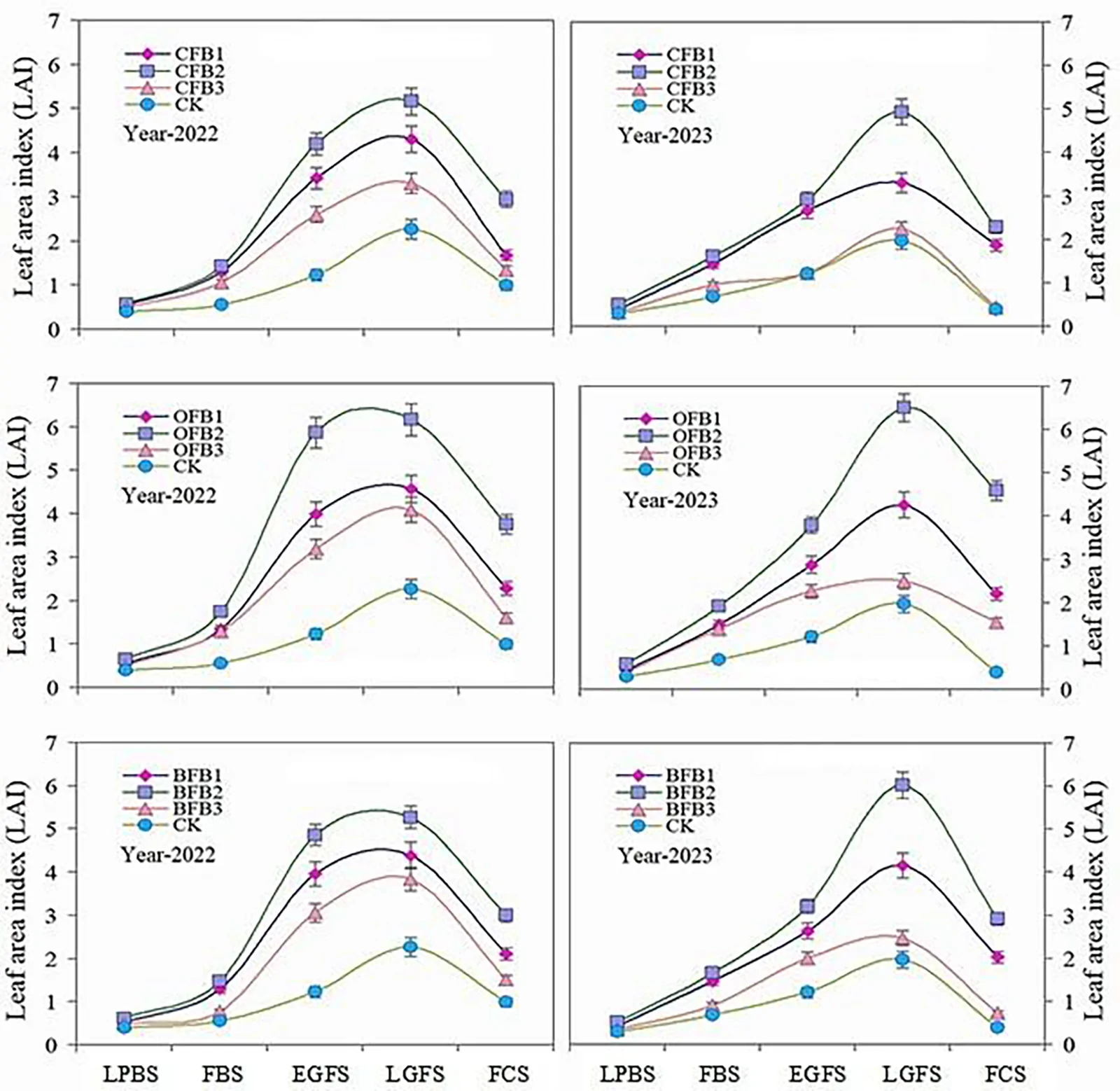
Effects of various treatments on the leaf area index (LAI) during various growth stages of blueberry under different foliar sprayers of the plant growth regulator brazide with integrated application of fertilization during the 2022 and 2023 study years. CFB1, chemical fertilizer with 1 µmol l^-1^ brazide; CFB2, chemical fertilizer with 3 µmol l^-1^ brazide; CFB3, chemical fertilizer with 5 µmol l^-1^ brazide; OFB1, organic fertilizer with 1 µmol l^-1^ brazide; OFB2, organic fertilizer with 3 µmol l^-1^ brazide; OFB3, organic fertilizer with 5 µmol l^-1^ brazide; BFB1, biofertilizer with 1 µmol l^-1^ brazide; BFB2, biofertilizer with 3 µmol l^-1^ brazide; BFB3, biofertilizer with 5 µmol l^-1^ brazide; CK, no fertilizer with water foliar spray; LPBS, Late pink bud stage; FBS, Full bloom stage; EGFS, Early green fruit stage; LGFS, Late green fruit stage; FCS, Fruit coloring stage. Vertical bars represent the (means ± SD) (n = 3).

As shown in **Table 2**, foliar application of the PGR brazide with fertilization affected nitrogen remobilization. Nitrogen remobilization efficiency in the B2 treatment was significantly higher than that in the B1 and B3 treatments, but the nitrogen contribution to fruit was higher in the OF treatment. Under the CF and BF treatments, increasing foliar application concentration of the brazide plant growth regulator (5 µmol L^-1^) caused a decrease in nitrogen uptake, the total N content at the FCS stage, and the contribution to fruit nitrogen. Nitrogen uptake in the B2 treatment was significantly higher than that in the B1 and B3 treatments. However, the nitrogen use efficiency and contribution to fruit nitrogen in the OFB2 treatment were higher than those of other fertilization and brazide plant growth regulator treatments. Under B1 and B3 treatments, nitrogen uptake was considerably greater in the CFB2 and OFB2 treatments than in other fertilization treatments; however, the total nitrogen content and the fruit nitrogen contribution in the OFB2 treatment during the FCS stage were considerably greater.

**Table 2:** Effects of different integrated applications of fertilization and plant growth regulator treatments on N remobilization and uptake and contribution to blueberry fruit N in the 2022 and 2023 study years.

Treatments	N remobilization (kg ha^-1^)			N update		
	Amount (kg ha^-1^)	Remobilization efficiency (%)	Contribution to fruit N (%)	Amount (kg ha^-1^)	Percentage in total N content (%)	Contribution to fruit N (%)
2022-Year						
CFB1	56.9d	28.1c	48.1c	84.8c	30.2c	51.9d
CFB2	71.2a	32.7b	54.6a	97.0a	36.7a	63.4a
CFB3	49.9f	23.3e	36.6e	59.2f	27.9d	44.0e
OFB1	63.7c	29.8c	48.5c	86.7c	31.7c	54.1c
OFB2	71.8a	36.3a	56.5a	99.0a	38.0a	65.5a
OFB3	53.1e	23.8	42.8d	73.9e	28.7d	44.8e
BFB1	56.5d	26.7d	43.5d	78.9d	29.7c	51.5d
BFB2	67.7b	31.7b	50.0b	89.8b	35.4b	57.2b
BFB3	43.8g	20.5f	35.6e	53.8g	26.7d	42.8e
CK	41.7g	20.1f	34.7e	44.2h	21.0e	38.4f
2023-Year						
CFB1	54.7d	26.7c	45.5d	77.7b	30.1c	54.5c
CFB2	64.9a	32.7b	51.6b	87.7a	36.0b	61.9b
CFB3	46.5e	23.3d	38.2e	58.4d	28.2d	48.4e
OFB1	58.2c	29.4b	48.0c	78.7b	31.7c	56.5c
OFB2	66.7a	40.7a	59.0a	86.0a	39.5a	68.0a
OFB3	49.7e	24.5d	39.5e	71.7c	28.7d	49.2e
BFB1	52.7d	24.9d	43.5d	73.4c	29.9c	52.0d
BFB2	62.1b	31.4b	50.8b	85.7a	35.1b	61.8b
BFB3	44.4f	21.7e	38.1e	52.0e	27.3d	46.9f
CK	35.9g	18.6e	32.1f	40.4f	20.5e	41.0g

CFB1, chemical fertilizer with 1 µmol l^-1^ brazide; CFB2, chemical fertilizer with 3 µmol l^-1^ brazide; CFB3, chemical fertilizer with 5 µmol l^-1^ brazide; OFB1, organic fertilizer with 1 µmol l^-1^ brazide; OFB2, organic fertilizer with 3 µmol l^-1^ brazide; OFB3, organic fertilizer with 5 µmol l^-1^ brazide; BFB1, biofertilizer with 1 µmol l^-1^ brazide; BFB2, biofertilizer with 3 µmol l^-1^ brazide; BFB3, biofertilizer with 5 µmol l^-1^ brazide; CK, no fertilizer with water foliar spray. Values are given as means, and different lowercase letters indicate significant differences at P ≤0.05 levels in the same line (LSD test) (n = 3).

Under the CF and OF treatments, NUE and NRE reached their maximum values, while NPFP decreased as the concentration of the plant growth regulator brazide increased from 3 µmol L^-1^ to 5 µmol L^-1^. NPFP was significantly higher under the OFB2 treatment than under other treatments (**Table 3**). Compared with the CK treatment, the NUE and NRE of the OFB2 and BFB2 treatments were considerably improved by 38.8% and 34.5%, and 31.8% and 31.9%, respectively. Compared with the CK treatment, the NPFP of the OFB2 and BFB2 treatments was considerably increased by 64.2% and 61.6%, respectively. In summary, these results highlight the impact of foliar application of the plant growth regulator brazide combined with fertilization on nitrogen fertilizer use in blueberry and are of great significance for optimizing nitrogen fertilizer use efficiency in blueberry production.

**Table 3:** Nitrogen fertilizer efficiency of blueberry under different integrated application of fertilization and plant growth regulator application strategies in 2022 and 2023.

Treatments	2022			2023		
	Nitrogen use efficiency (kg kg^-1^)	Nitrogen recovery efficiency (%)	Nitrogen partial productivity (kg kg^-1^)	Nitrogen use efficiency (kg kg^-1^)	Nitrogen recovery efficiency (%)	Nitrogen partial productivity (kg kg^-1^)
CFB1	5.43c	27.12c	46.62e	7.20c	27.81c	42.42f
CFB2	6.74b	32.25a	65.99c	9.36a	29.23b	61.77c
CFB3	4.69d	24.41d	32.91h	6.68d	25.62d	29.98h
OFB1	6.23b	29.79b	59.54d	8.13b	28.92b	49.54d
OFB2	7.83a	32.97a	88.87a	9.72a	31.92a	80.53a
OFB3	5.07c	26.19c	45.76f	6.82d	27.15c	40.75f
BFB1	6.05b	27.26c	48.51e	7.57c	28.42b	44.55e
BFB2	7.79a	32.44a	84.71b	9.69a	29.91b	73.21b
BFB3	5.02c	26.58c	43.03g	6.73d	26.03c	39.71g
CK	--	--	--	--	--	--

CFB1, chemical fertilizer with 1 µmol l^-1^ brazide; CFB2, chemical fertilizer with 3 µmol l^-1^ brazide; CFB3, chemical fertilizer with 5 µmol l^-1^ brazide; OFB1, organic fertilizer with 1 µmol l^-1^ brazide; OFB2, organic fertilizer with 3 µmol l^-1^ brazide; OFB3, organic fertilizer with 5 µmol l^-1^ brazide; BFB1, biofertilizer with 1 µmol l^-1^ brazide; BFB2, biofertilizer with 3 µmol l^-1^ brazide; BFB3, biofertilizer with 5 µmol l^-1^ brazide; CK, no fertilizer with water foliar spray. Values are given as means, and different lowercase letters indicate significant differences at P ≤0.05 levels in the same line (LSD test) (n = 3).

### 3.2 Net photosynthetic rate and lodging rate

At the LPBS, FBS, and FCS stages of blueberry, the Pn value significantly increased with increasing concentrations of the plant growth regulator brazide (3 µmol l^-1^) (**Figure 2**). Under the same treatments in both study years, the Pn value of blueberry significantly increased from the LPBS to the FBS stage but rapidly decreased from the FBS to the FCS stage (**Figure 2**). At a concentration of 3 µmol L^-1^ of the plant growth regulator brazide, the average Pn value of the OF treatment was considerably greater than that of all other treatments. At the LPBS, FBS, and FCS stages in both study years, the Pn value of CFB2 was significantly higher compared with the CFB1, CFB3, and CK treatments. There were non-significant variances in Pn values ​​between CFB3 and CK at different growth stages. At each growth stage, the effect of OF on the Pn value of blueberry was considerably greater than that of other treatments at all concentrations of the plant growth regulator brazide.

**Figure 2:**
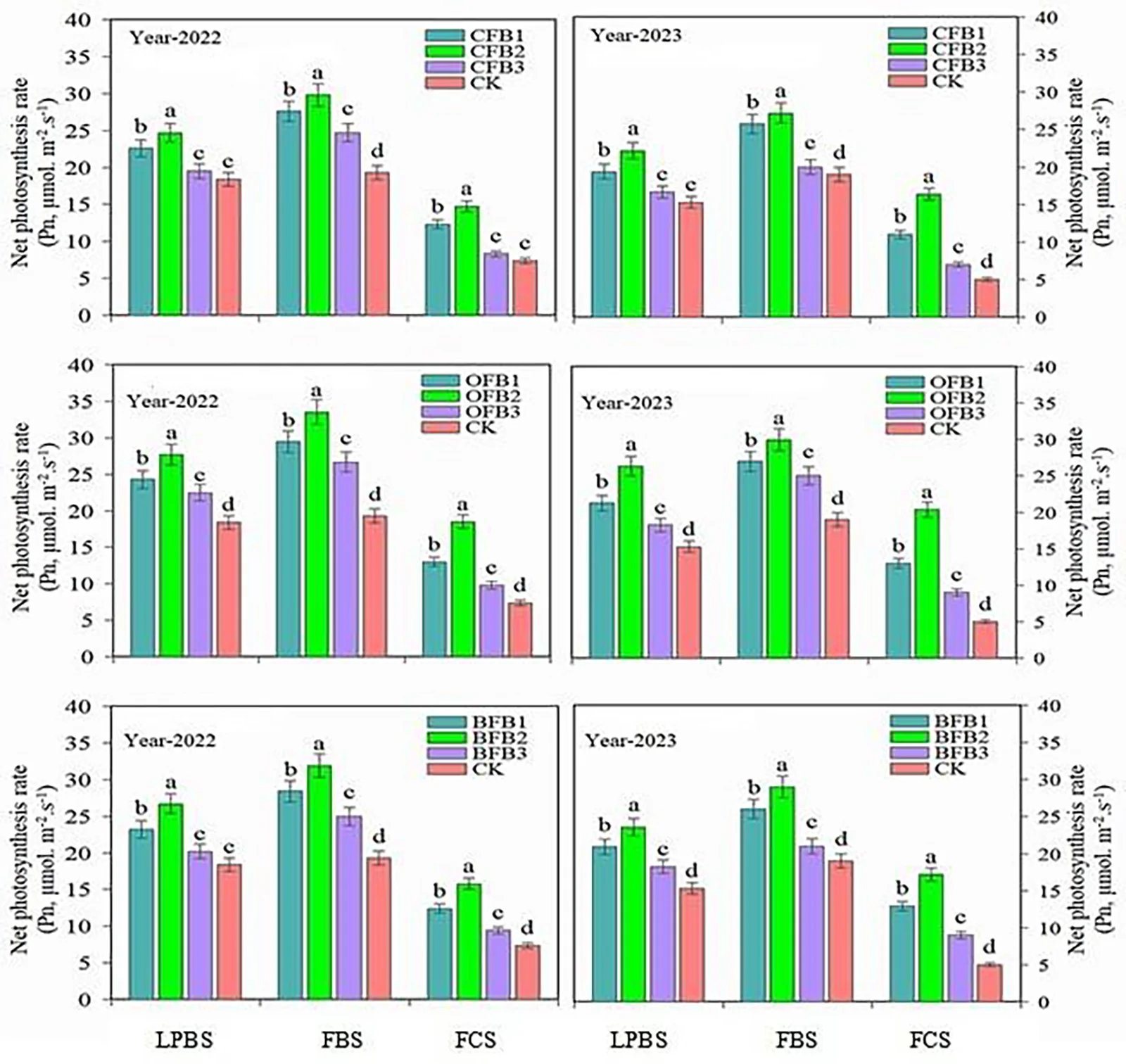
Effects of various treatments on the net photosynthesis rate at various growth stages of blueberry under different foliar sprayers of the plant growth regulator brazide with integrated application of fertilization during the 2022 and 2023 study years. CFB1, chemical fertilizer with 1 µmol l^-1^ brazide; CFB2, chemical fertilizer with 3 µmol l^-1^ brazide; CFB3, chemical fertilizer with 5 µmol l^-1^ brazide; OFB1, organic fertilizer with 1 µmol l^-1^ brazide; OFB2, organic fertilizer with 3 µmol l^-1^ brazide; OFB3, organic fertilizer with 5 µmol l^-1^ brazide; BFB1, biofertilizer with 1 µmol l^-1^ brazide; BFB2, biofertilizer with 3 µmol l^-1^ brazide; BFB3, biofertilizer with 5 µmol l^-1^ brazide; CK, no fertilizer with water foliar spray; LPBS, Late pink bud stage; FBS, Full bloom stage; FCS, Fruit coloring stage. Vertical bars represent the (means ± SD) (n = 3).

In both study years, the lodging resistance index of the OFB3 treatment was significantly lower than that of all other treatments, but considerably greater than that of the CFB1 and CK treatments (**Figure 3**). Compared with the CFB1 treatment, the mean lodging rates of the OFB3 and BFB3 were considerably lower in both study years; moreover, the OFB3 treatment had the lowest lodging rate. Compared with the CK treatment, in both study years, the combined application of a high concentration of the plant growth regulator brazide (5 µmol L^-1^) and organic fertilizer significantly reduced the lodging resistance index, but the difference remained statistically significant. Therefore, regardless of fertilizer application ratio, in both study years, the high concentration of the plant growth regulator brazide (5 µmol L^-1^) resulted in a lower lodging resistance index and lodging rate than the low concentration of brazide in the B1 and B2 treatments (**Figure 4**). Compared with the OF treatment, the lodging resistance index of the CF and BF treatments was considerably improved in the 2022 and 2023 study years. This resulted in a significantly lower lodging rate for the OF treatment compared with the CF and BF treatments.

**Figure 3:**
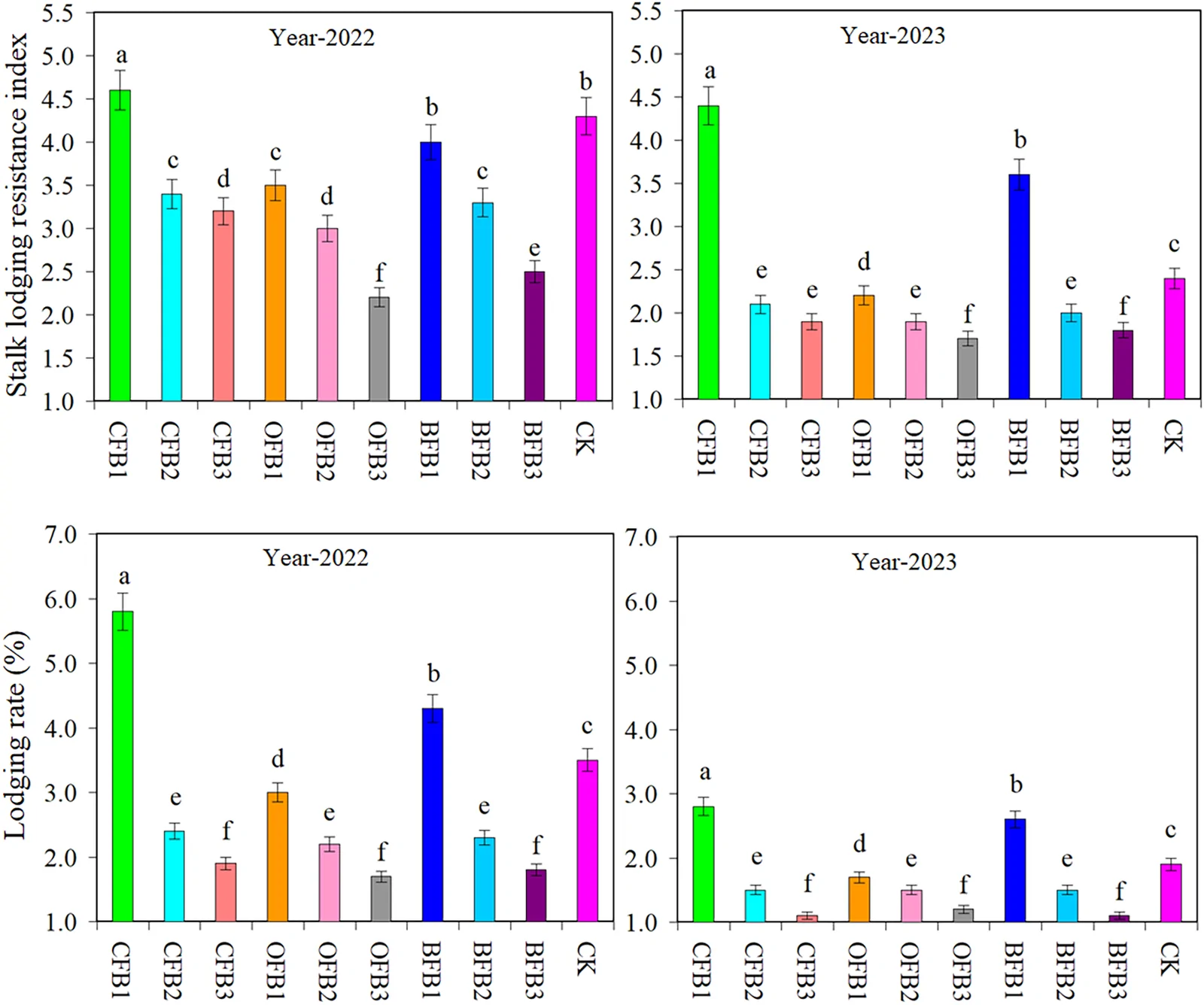
Effects of various treatments on the stalk lodging resistance index (%) and lodging rate (%) of blueberry under different foliar sprayers of the plant growth regulator brazide with integrated application of fertilization during the 2022 and 2023 study years. CFB1, chemical fertilizer with 1 µmol l^-1^ brazide; CFB2, chemical fertilizer with 3 µmol l^-1^ brazide; CFB3, chemical fertilizer with 5 µmol l^-1^ brazide; OFB1, organic fertilizer with 1 µmol l^-1^ brazide; OFB2, organic fertilizer with 3 µmol l^-1^ brazide; OFB3, organic fertilizer with 5 µmol l^-1^ brazide; BFB1, biofertilizer with 1 µmol l^-1^ brazide; BFB2, biofertilizer with 3 µmol l^-1^ brazide; BFB3, biofertilizer with 5 µmol l^-1^ brazide; CK, no fertilizer with water foliar spray. Vertical bars represent the (means ± SD) (n = 3).

**Figure 4:**
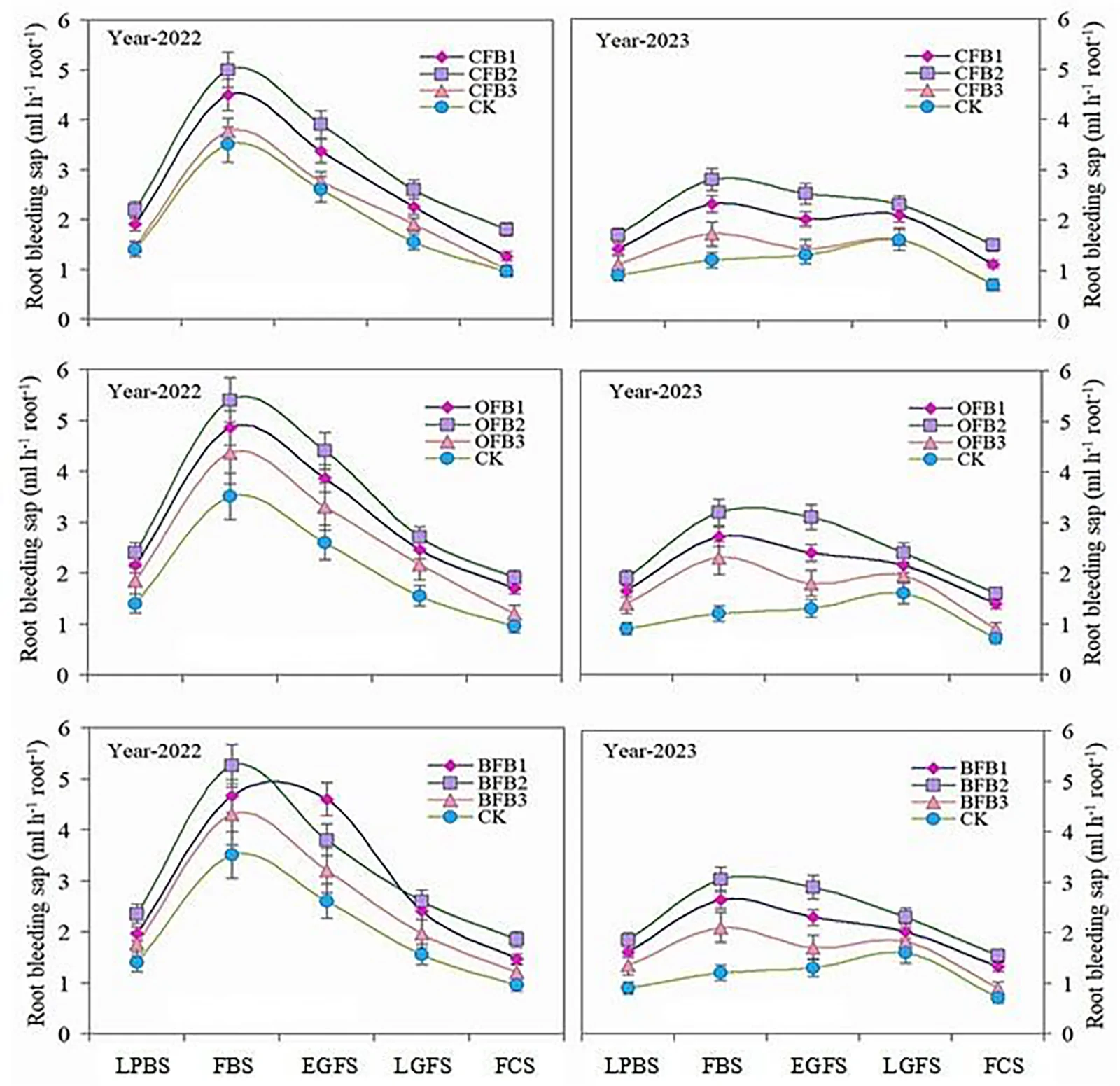
Effects of various treatments on root bleeding sap at various growth stages of blueberry under different foliar sprays of the plant growth regulator brazide with integrated application of fertilization during the 2022 and 2023 study years. CFB1, chemical fertilizer with 1 µmol l^-1^ brazide; CFB2, chemical fertilizer with 3 µmol l^-1^ brazide; CFB3, chemical fertilizer with 5 µmol l^-1^ brazide; OFB1, organic fertilizer with 1 µmol l^-1^ brazide; OFB2, organic fertilizer with 3 µmol l^-1^ brazide; OFB3, organic fertilizer with 5 µmol l^-1^ brazide; BFB1, biofertilizer with 1 µmol l^-1^ brazide; BFB2, biofertilizer with 3 µmol l^-1^ brazide; BFB3, biofertilizer with 5 µmol l^-1^ brazide; CK, no fertilizer with water foliar spray; LPBS, Late pink bud stage; FBS, Full bloom stage; EGFS, Early green fruit stage; LGFS, Late green fruit stage; FCS: Fruit coloring stage. Vertical bars represent the (means ± SD) (n = 3).

### 3.3 Soil respiration rate and physiological activities of blueberry leaves

During the 2-year field experiment, the OFB2 treatment, which included a medium concentration of the plant growth regulator brazide (3 μmol L^-1^), significantly influenced soil respiration rates at different blueberry growth stages (**Table 4**). In both 2022 and 2023, notable variations in soil respiration were observed during the late pink bud, full bloom, and fruit coloration stages under the OFB2 treatment. In contrast, the BFB1 and OFB3 treatments did not significantly affect soil respiration across the different growth stages in either experimental year. Overall, the combined application of integrated fertilization strategies and a medium concentration of brazide (3 μmol L^-1^) enhanced soluble protein, soluble sugar, superoxide dismutase (SOD), malondialdehyde (MDA), and chlorophyll contents compared with the CK treatment. Among all treatments, OFB2 markedly promoted the accumulation of soluble sugars, soluble proteins, SOD activity, MDA, and chlorophyll content in blueberry leaves. However, the application of chemical fertilizer combined with a higher brazide concentration (5 μmol L^-1^) reduced soluble sugar, soluble protein, SOD, and MDA levels in the leaves (**Table 5**). These findings indicate that excessive brazide application may negatively affect physiological activity in blueberry plants. Integrated fertilization treatments generally resulted in greater soluble sugar and protein accumulation than the CK treatment, with the OFB2 treatment showing the highest values. This suggests that the combined use of organic fertilizer and a moderate brazide concentration effectively enhanced carbohydrate accumulation in blueberry leaves. In addition, SOD activity and MDA content were highest under the OFB2 and BFB2 treatments. Across both study years, chlorophyll content was consistently higher under OFB2, OFB1, and BFB2 treatments than under the other treatments.

**Table 4:** Effects of different integrated application of fertilization and plant growth regulator application strategies on soil respiration rate (µmol m^-2^ s^-1^) of blueberry in the 2022 and 2023 study years.

Treatments	Soil respiration rate (µmol m^-2^ s^-1^)		
	Late pink bud stage (LPBS)	Full bloom stage (FBS)	Fruit coloring stage (FCS)
2022-Year			
CFB1	2.89d	2.50c	2.28c
CFB2	4.08c	2.90b	2.19c
CFB3	2.28d	1.80e	1.19e
OFB1	4.09b	2.49c	2.67b
OFB2	4.76a	3.73a	3.17a
OFB3	3.27c	2.22d	1.64d
BFB1	3.26c	2.01d	1.62d
BFB2	4.48a	2.93b	2.89b
BFB3	2.78d	1.78e	1.46d
CK	1.78e	1.30f	1.09f
2023-Year			
CFB1	3.89e	3.19d	2.40d
CFB2	4.54c	4.11c	3.23c
CFB3	2.78f	2.80e	1.99e
OFB1	4.87c	4.33c	4.11a
OFB2	5.12a	4.73a	4.64a
OFB3	3.70e	3.20d	2.10d
BFB1	4.33d	3.25d	2.04e
BFB2	4.96b	4.59b	3.82b
BFB3	2.99f	2.47e	1.69f
CK	2.49g	1.97f	1.39f

CFB1, chemical fertilizer with 1 µmol l-1 brazide; CFB2, chemical fertilizer with 3 µmol l-1 brazide; CFB3, chemical fertilizer with 5 µmol l-1 brazide; OFB1, organic fertilizer with 1 µmol l-1 brazide; OFB2, organic fertilizer with 3 µmol l-1 brazide; OFB3, organic fertilizer with 5 µmol l-1 brazide; BFB1, biofertilizer with 1 µmol l-1 brazide; BFB2, biofertilizer with 3 µmol l-1 brazide; BFB3, biofertilizer with 5 µmol l-1 brazide; CK, no fertilizer with water foliar spray. LPBS, Late pink bud stage; FBS, Full bloom stage; FCS, Fruit coloring stage. Values are given as means, and different lowercase letters indicate significant differences at P ≤0.05 levels in the same line (LSD test) (n = 3).

**Table 5:** Effects of different integrated application strategies of fertilization and plant growth regulator application strategies on the physiological activity of blueberry in the 2022 and 2023 study years.

Treatments	Soluble protein (%)	Soluble sugar (%)	SOD (μg g^-1^)	MDA (nmol g^-1^)	Chlorophyll (mg g^-1^)
2022-Year					
CFB1	5.9c	3.3b	120.9d	51.9e	4.4a
CFB2	6.6b	3.5a	139.7c	56.6d	3.6b
CFB3	4.7d	3.0b	110.7e	44.7f	2.2c
OFB1	6.4b	3.4a	158.7b	59.2c	4.5a
OFB2	7.0a	3.8a	179.6a	68.9a	4.8a
OFB3	5.4c	3.1b	114.7e	46.2f	3.5b
BFB1	6.4b	3.4a	142.6c	55.6d	3.7b
BFB2	6.6b	3.8a	175.1a	66.8b	4.6a
BFB3	5.0c	3.4a	129.7d	52.7e	3.4b
CK	4.1d	2.6c	108.9e	43.8f	1.9d
2023-Year					
CFB1	5.6b	3.4b	119.9e	50.9d	3.5b
CFB2	6.1a	3.5b	141.6c	54.6c	3.5b
CFB3	4.4c	3.2b	109.7f	43.7e	1.3d
OFB1	6.1a	3.5b	157.7b	58.2b	3.6a
OFB2	6.7a	3.9a	178.6a	67.9a	3.9a
OFB3	5.1b	3.2b	113.7f	45.2e	3.5b
BFB1	5.8b	3.6a	138.7c	55.6c	2.6c
BFB2	6.3a	3.9a	174.1a	65.8a	3.7a
BFB3	4.7c	3.5b	128.7d	51.7d	2.5c
CK	3.8d	2.8c	107.9f	42.8f	1.0e

CFB1, chemical fertilizer with 1 µmol l-1 brazide; CFB2, chemical fertilizer with 3 µmol l-1 brazide; CFB3, chemical fertilizer with 5 µmol l-1 brazide; OFB1, organic fertilizer with 1 µmol l-1 brazide; OFB2, organic fertilizer with 3 µmol l-1 brazide; OFB3, organic fertilizer with 5 µmol l-1 brazide; BFB1, biofertilizer with 1 µmol l-1 brazide; BFB2, biofertilizer with 3 µmol l-1 brazide; BFB3, biofertilizer with 5 µmol l-1 brazide; CK, no fertilizer with water foliar spray. Values are given as means, and different lowercase letters indicate significant differences at P ≤0.05 levels in the same line (LSD test) (n = 3).

### 3.4 Blueberry stems compositional traits

This research suggests that under integrated fertilization conditions, foliar application of the plant growth regulator brazide can better optimize the blueberry canopy structure. The results showed that foliar application of the plant growth regulator brazide combined with integrated fertilization affected the stem compositional traits of the blueberry stem. Differences in stem quality were observed between the B1 and B3 treatments in both study years. The crushing strength (CS), hemicellulose content (HC), cellulose content (CC), and lignin content (LC) in the OFB2 treatment were considerably greater than those of all other treatments (**Table 6**). Foliar application of 3 µmol L^-1^ of the plant growth regulator brazide combined with the OF treatment affected the stem CS, HC, CC, and LC of blueberry stems. Under various integrated fertilizer management strategies, foliar application of the plant growth regulator brazide affected GHGI emissions. Compared with the OFB2 and OFB1 treatments, the CK and CFB3 treatments increased GHGI by 57.0% and 74.6%, respectively, and 38.0% and 63.4%, respectively (**Table 7**). The increase in GHGI was primarily attributed to elevated greenhouse gas emissions.

**Table 6:** Effects of different integrated application of fertilization and plant growth regulator application strategies on quality of blueberry stems in the 2022 and 2023 study years.

Treatments	Crushing strength (CS, N cm^-2^)	Hemicellulose content (HC, mg g^-1^)	Lignin content (LC, mg g^-1^)	cellulose content (CC, mg g^-1^)
2022-Year				
CFB1	462.0f	177.2c	290.1c	23.1c
CFB2	548.8c	189.0b	302.1b	26.1b
CFB3	442.3g	173.3d	285.4e	22.1c
OFB1	532.0d	185.7b	301.9b	25.6b
OFB2	621.8a	198.9a	320.8a	30.8a
OFB3	508.4e	179.8c	292.2c	22.5c
BFB1	481.8e	180.5c	295.4c	23.5c
BFB2	616.0b	197.7a	308.6b	27.9b
BFB3	457.4f	177.0c	288.0d	22.4c
CK	431.2h	168.5e	284.2	20.3c
2023-Year				
CFB1	462.2d	187.9b	315.3b	23.6c
CFB2	494.3b	190.4a	319.8b	27.2b
CFB3	454.3e	184.0c	312.9c	22.3d
OFB1	494.2b	190.1a	318.4b	26.6b
OFB2	536.2a	193.5a	325.3a	32.0a
OFB3	478.4c	186.1b	315.9b	23.8c
BFB1	467.8d	188.5b	316.0b	24.0c
BFB2	522.2a	192.4a	324.4a	31.3a
BFB3	459.0e	184.9c	313.8c	22.7d
CK	433.6f	179.0d	305.2d	21.2d

CFB1, chemical fertilizer with 1 µmol l-1 brazide; CFB2, chemical fertilizer with 3 µmol l-1 brazide; CFB3, chemical fertilizer with 5 µmol l-1 brazide; OFB1, organic fertilizer with 1 µmol l-1 brazide; OFB2, organic fertilizer with 3 µmol l-1 brazide; OFB3, organic fertilizer with 5 µmol l-1 brazide; BFB1, biofertilizer with 1 µmol l-1 brazide; BFB2, biofertilizer with 3 µmol l-1 brazide; BFB3, biofertilizer with 5 µmol l-1 brazide; CK, no fertilizer with water foliar spray. Values are given as means, and different lowercase letters indicate significant differences at P ≤0.05 levels in the same line (LSD test) (n = 3).

**Table 7:** Effects of different treatments^a^ on the number of flower buds, number of new shoots, total blueberry yield (t ha^-1^), greenhouse gas intensity (GHGI), evapotranspiration (ET, mm), and water use efficiency (WUE, kg ha^-1^ mm^-1^), and net profit of blueberry during 2022 and 2023.

Treatments	No. of flowers buds	No. of new shoots	Total blueberry yield (t ha^-1^)	GHGI (kg CO_2_-eq kg^-1^ blueberry)	ET (mm)	WUE (kg ha^-1^ mm^-1^)	Net profit (CNY ha^-1^)
2022-Year							
CFB1	897d	50c	9.2e	1.24f	391c	23.5f	269848f
CFB2	960c	52c	13.8d	2.07c	461a	29.9e	407380d
CFB3	784g	35e	7.0f	2.45b	361d	19.4g	202945i
OFB1	985b	62b	14.7c	0.97g	374d	39.3c	433511c
OFB2	1041a	68a	20.5a	1.66d	399c	51.4a	609568a
OFB3	816f	38e	8.1e	1.85d	329f	24.6f	237100g
BFB1	833e	45d	13.2d	1.00f	372d	35.5d	389665e
BFB2	989b	63b	19.2b	1.67e	423b	45.4b	569231b
BFB3	867d	41d	7.8g	2.29b	343e	22.7f	228568h
CK	718h	31f	6.8h	3.38a	316g	21.5g	198100j
2023-Year							
CFB1	830e	53e	11.4f	1.57e	441d	25.9d	335848f
CFB2	974b	67c	13.5d	2.46c	505a	26.7d	398380d
CFB3	798f	50e	6.4i	3.07b	360h	17.8f	184945i
OFB1	1003a	78b	18.6b	1.05f	384f	48.4a	550511b
OFB2	1055a	83a	20.3a	1.76e	474c	42.8b	603568a
OFB3	911c	65c	9.2g	1.95d	344i	26.7d	270100g
BFB1	847e	60d	12.5e	1.04f	415e	30.1c	368665e
BFB2	999b	77b	14.4c	2.07d	484b	29.8c	425231c
BFB3	881d	56e	7.4h	3.19b	350j	21.1e	216568h
CK	732g	46f	5.9j	4.58a	331k	17.8f	171100j

CFB1, chemical fertilizer with 1 µmol l-1 brazide; CFB2, chemical fertilizer with 3 µmol l-1 brazide; CFB3, chemical fertilizer with 5 µmol l-1 brazide; OFB1, organic fertilizer with 1 µmol l-1 brazide; OFB2, organic fertilizer with 3 µmol l-1 brazide; OFB3, organic fertilizer with 5 µmol l-1 brazide; BFB1, biofertilizer with 1 µmol l-1 brazide; BFB2, biofertilizer with 3 µmol l-1 brazide; BFB3, biofertilizer with 5 µmol l-1 brazide; CK, no fertilizer with water foliar spray. Values are given as means, and different lowercase letters indicate significant differences at P ≤0.05 levels in the same line (LSD test) (n = 3).

### 3.5 Blueberry production and net profit

In both study years, foliar application of the plant growth regulator brazide combined with fertilization affected the number of flower buds and new shoots, evapotranspiration, blueberry yield, WUE, and net profit (**Table 7**). Compared with CK, the average blueberry yield under the OFB2 treatment increased significantly by 68.9% over 2 years; however, compared with B2, the blueberry yield under the B1 and B3 treatments was considerably decreased. The OFB2 treatment produced the greatest number of flower buds, whereas the lowest number was recorded under the CK treatment. Therefore, the combined application of organic fertilizer and a moderate concentration of the plant growth regulator brazide (3 μmol L^-1^) significantly enhanced the formation of flower buds and the growth of new shoots in blueberry plants (**Table 7**). The results indicate that, under different concentrations of the plant growth regulator brazide applied through foliar spraying, the evapotranspiration (ET) of CFB2 was considerably greater than that of the OF and BF treatments, but there was a non-significant variance between the CFB1 and OFB2 treatments. The 2-year average data showed that the WUE of the OFB2 treatment was 58.2% greater than that of the CK, but the net yield of B1 was lower than that of the B3 treatment. Overall, although the OF treatment had the highest blueberry yield under the B2 treatment, it had lower evapotranspiration, and its net profit and NUE were higher than those of all other treatments. More importantly, in semi-arid regions, the OF treatment under the B2 treatment had the highest (606,568 CNY ha^-1^) net profit.

## 4 Discussion

Maintaining a high photosynthetic rate is crucial for plant growth and energy metabolism, especially under water conditions, when photosynthesis is the most sensitive physiological process (Janeczko et al., 2016; Zhu et al., 2017). Exogenous supply of brazide can enhance photosynthesis by protecting photosynthetic organs from oxidative toxicity under water conditions (Li et al., 2020; Jabborova et al., 2025). At different growth stages, the net photosynthetic rate (Pn) of blueberry significantly increased with increasing concentrations of the plant growth regulator brazide (3 µmol L^-1^). The Pn values ​​of CFB2 were considerably greater than those of CFB1, CFB3, and CK at the LPBS, FBS, and FCS stages. At all growth stages, the effect of the OF treatment on blueberry Pn values ​​was significantly higher than that of the other treatments across all brazide concentrations. This finding is consistent with earlier research indicating that the application of exogenous brazide can improve photosynthetic characteristics under water stress (Chen et al., 2012; Behnamnia, 2015). The effect of brazide on stomatal movement depends on its concentration; low concentrations of brazide promote stomatal opening, while high concentrations induce stomatal closure (Xia et al., 2021; Jabborova et al., 2025). Blueberry growth and yield are affected by the combined application of integrated fertilizers. Zuo et al. (2024) found that reducing fertilizer application led to a considerable decrease in blueberry yield and LAI. Li et al. (2020) reported that applying higher concentrations of organic fertilizer could increase LAI and blueberry yield. LAI generally increases with increasing concentration of brazide, reaching a maximum at 3 µmol L^-1^. During the growth stages of EGFS, LGFS, and FCS, the LAI of treatments B1 and B3 was lower than that of treatment B2; during the growth stage R3, the average LAI of treatments B3 and B1 was significantly lower than that of treatment OFB2 in both study years. LAI is an important indicator reflecting the performance of the plant canopy structure, reflecting the plant’s ability to intercept radiation and photosynthesize (Braha and Kullaj, 2024; Guo et al., 2022), and water and fertilizer management affect LAI (Messiga et al., 2018; Zhai et al., 2023). Under different foliar spraying concentrations of the plant growth regulator brazide, we found no significant differences in LAI and Pn among the fertilization treatments, while the CK treatment had significantly lower LAI at all growth stages than other fertilization treatments. These results are consistent with those of Li et al. (2020) and Messiga et al. (2020), who found that water deficiency reduces LA and chlorophyll, accelerates senescence, and impairs photosynthetic capacity. Fertilizer significantly affects LAI and Pn in blueberry (Wang et al., 2024; Poorter et al., 2009).

Chemical fertilizers can provide sufficient inorganic nitrogen for direct plant uptake. Furthermore, treatment with chemical fertilizers (CFB) containing plant growth-promoting rhizobacteria can encourage plant development by improving nutrient availability and nitrogen fixation (Hernandez et al., 2015; Wang et al., 2018). Under the B2 treatment, the nitrogen remobilization efficiency was considerably higher than that in the B1 and B3 treatments (both treated with the plant growth regulator brazide via foliar spraying). However, under conditions of combined application of organic fertilizers, the nitrogen remobilization efficiency and its contribution to fruit nitrogen were both improved. In the later stages, chemical fertilizer and biofertilizer (CFOB), under suitable moisture and temperature conditions, can enhance the activity of soil microorganisms, as well as available soil nutrients, ultimately promoting plant growth and photosynthesis, consistent with the findings of Zheng et al. (2017). Fruit nitrogen accumulation is the result of NUE and nitrogen uptake (Li et al., 2020; Petridis et al., 2018). Previous research has shown that nitrogen remobilization efficiency and nitrogen uptake decrease with decreasing PGR levels (Díaz-Pérez et al., 2021; Jabborova et al., 2025). However, our study found that under combined CF and BF fertilization conditions, with increasing foliar application of brazide (at a concentration of 5 µmol L^-1^), nitrogen uptake, total N content, and the contribution to fruit nitrogen all showed a decreasing trend. The nitrogen uptake in B2 was considerably greater than that in B1 and B3, which is consistent with the findings of Zhang et al. (2024).

Among the various characteristics of blueberry stems, stem crushing strength (CS), hemicellulose content (HC), cellulose content (CC), and lignin content (LC) are the most important indicators affecting the lodging index (Gao et al., 2020; Guo et al., 2022). In the OF treatment, when the concentration of brazide was increased from 1 µmol l^-1^ to 3 µmol l^-1^, morphological characteristics increased significantly. Blueberry stem structure affects the quality of the blueberry stem (Tian et al., 2019; Messiga et al., 2021). The OFB2 treatment had significantly higher CS, LC, HC, and CC than those of the control (CK). Increased cellulose and lignin content, as well as increased stem diameter and wall thickness, can enhance stem strength and reduce the risk of lodging (Feng et al., 2023a). However, excessive nitrogen fertilizer use can impede stem development and reduce lignification, weakening stem strength (Luo et al., 2019; Anderson et al., 2023). Changes in blueberry morphological characteristics and improved stem quality can help reduce the risk of lodging (Wu et al., 2023). The OFB2 treatment forms a better blueberry canopy structure and increases the photosynthetic rate (Pn) (Zheng et al., 2017; Wei et al., 2018). Improving the physical strength and morphological characteristics of blueberry stems is a major goal for increasing stem lodging resistance (Okuno et al., 2014). Compared with the control (CK), B1, and B3 treatments, treatment B2 with fertilizer containing CF, OF, and BF considerably improved the bleeding sap. Improving the stem morphological characteristics of rice significantly improves lodging resistance in this plant (Zheng et al., 2017). In addition, the stem bending resistance of blueberry stems was highest in the OFB2 treatment, followed by the CFB2 and BFB2 treatments. In blueberry, a reduced lodging rate and improved photosynthetic performance of leaves contributed to increased blueberry yield (Dong et al., 2022). Lodging stress is an important constraint that reduces blueberry yield and quality worldwide (Ma et al., 2018; Sekhon et al., 2020). High lignin accumulation in the blueberry stem can enhance lodging resistance (Feng et al., 2023b).

Crop yield, evapotranspiration, and crop nitrogen accumulation affect WUE, NRE, and NUE (Zhai et al., 2022). Our results show that WUE, NRE, and NUE all initially improved and then decreased with decreasing fertilizer level, consistent with the findings of Li et al. (2020). Compared with the CK treatment, the OFB2 treatment considerably improved blueberry yield by 68.9%; however, compared with the B2 treatment, the B1 and B3 treatments showed considerably lower blueberry yield. These results indicate that under different concentrations of the plant growth regulator brazide applied as a foliar spray, the evapotranspiration of CFB2 was considerably greater. Both the CF and OF treatments achieved maximum NUE and WUE, while NPFP decreased as the concentration of the plant growth regulator brazide increased from 3 µmol L^-1^ to 5 µmol L^-1^, with the OFB2 treatment showing significantly higher NPFP values ​​than the other treatments. Our results showing that the application of PGR can increase blueberry yield and reduce lodging rate, and this is consistent with previous findings (Kuai et al., 2015; Wang et al., 2015). Compared with the OFB2 and OFB1 treatments, the CK and CFB3 treatments significantly increased GHGI, respectively. The increase in GHGI was primarily attributed to elevated greenhouse gas emissions, which were mainly due to the crop’s inability to effectively utilize nutrients, to reduced blueberry yield (Ning et al., 2022). Overall, although the OF treatment under B2 conditions had the highest blueberry yield, it also had lower evapotranspiration, higher water and fertilizer use efficiency, and a higher effective utilization rate compared to other treatments. More importantly, in semi-arid regions, the OF treatment under B2 conditions had the highest net profit (606,568 CNY ha^-1^). Although factors such as blueberry yield, NUE, economic benefits, and environmental impact can be considered to formulate relatively reasonable plant growth regulator brazide and fertilization strategies (Wang et al., 2024), further research is needed.

## 5 Conclusion

The combined application of biofertilizers, chemical fertilizers, and organic fertilizers under brazide regulation improved blueberry growth, lodging resistance, and resource use efficiency. The OFB2 treatment improved stem morphological and mechanical characteristics, resulting in stronger lodging resistance and better plant stability. The OFB2 treatment promoted photosynthetic performance, root development, and nutrient accumulation, improving water and nitrogen use efficiencies as well as nitrogen uptake. Consequently, the OFB2 treatment significantly increased blueberry yield, WUE, and economic returns. These findings demonstrate the potential of brazide-based integrated nutrient management as an effective strategy and offer new insights for improving blueberry productivity; therefore, it is important to adjust fertilizer nitrogen management with various PGRs according to plant needs to maintain long-term productivity. Further investigations are needed to explore optimal fertilizer, irrigation strategies, and control mechanisms under multi-location and multi- blueberry cultivars. Additionally, further studies should consider extending the application of various PGRs, which limits the risk of soil nitrogen accumulation and nitrogen leakage into aquifers of blueberry farms under different irrigation management strategies.

## Data Availability

The original contributions presented in the study are included in the article/supplementary material. Further inquiries can be directed to the corresponding author.

## References

[B1] Ali S. Khan N. (2021). Delineation of mechanistic approaches employed by plant growth promoting microorganisms for improving water stress tolerance in plants. Microbiol. Res. 249, 126771. doi: 10.1016/j.micres.2021.126771 33930840

[B2] Ali S. Xu Y. Y. Ma X. C. Ahmad I. Manzoor Jia Q. M. . (2019). Deficit irrigation strategies to improve winter wheat productivity and regulating root growth under different planting patterns. Agric. Water Manage. 219, 1–11. doi: 10.1016/j.agwat.2019.03.03842574925

[B3] Anderson T. Hummer K. E. Luby C. (2023). Heat stress on northern highbush blueberry in Oregon and Washington, USA. Acta Hortic. 1381, 115–122. doi: 10.17660/actahortic.2023.1381.16

[B4] Bañados M. P. Strik B. C. Bryla D. R. Righetti T. L. (2012). Response of highbush blueberry to nitrogen fertilizer during field establishment, I: Accumulation and allocation of fertilizer nitrogen and biomass. HortScience 47, 648–655.

[B5] Bañados P. Strik B. Righetti T. (2006). The uptake and use of ^15^N-nitrogen in young and mature field-grown highbush blueberries. Acta Hortic. 715, 357–364.

[B6] Behnamnia M. (2015). Protective roles of brassinolide on tomato seedlings under water stress. Int. J. Agric. Crop Sci. 8, 552–559.

[B8] Braha S. Kullaj E. (2024). The effect of different methods of fertilization with ammonium sulfate and urea on yield and fruit size of highbush blueberry (*Vaccinium corymbosum* L.). Ecol. Eng. Environ. Technol. 25, 116–124. doi: 10.12912/27197050/183169 27241809 PMC4970344

[B9] Bryla D. R. Strik B. C. Bañados M. P. Righetti T. L. (2012). Response of high bush blueberry to nitrogen fertilizer during field establishment, II: Plant nutrient requirements in relation to nitrogen fertilizer supply. HortScience 47, 917–926. doi: 10.21273/hortsci.47.7.917

[B10] Chen H. Zhao J. Jiang J. Zhao Z. Guan Z. Chen S. . (2021). Effects of inorganic, organic and bio-organic fertilizer on growth, rhizosphere soil microflora and soil function sustainability in chrysanthemum monoculture. Agriculture 11, 1214. doi: 10.3390/agriculture1112121430654563

[B11] Chen W. R. Zeng W. W. Li Y. X. (2012). Physiological response to water stress and comprehensive evaluation of water resistance of tall blueberries. J. Hort 39, 637–646.

[B12] Chen X. G. Shi C. Y. Yin Y. P. Wang Z. L. Shi Y. H. Peng D. L. . (2011). Relationship between lignin metabolism and lodging resistance in wheat. Acta Agron. Sin. 37, 1616–1622. doi: 10.3724/SP.J.1006.2011.01616

[B13] Cheng Y. Luo M. Zhang T. G. Yan S. H. Wang C. Feng H. . (2023). Organic substitution improves soil structure and water and nitrogen status to promote sunflower (*Helianthus annuus* L.) growth in an arid saline area. Agr. Water Manage. 283, 108320. doi: 10.1016/j.agwat.2023.10832042574925

[B14] China Agriculture Yearbook Editorial Board (2014). China Agriculture Yearbook 2014 (Beijing: China Agriculture Publishing House), 44–48.

[B15] Díaz-Pérez J. C. da Silva A. L. B. R. Valdez-Aguilar L. A. (2021). Seasonal plant growth, leaf and bulb mineral nutrients, and bulb yield and quality under chemical, mixed, and organic fertilization in sweet onion (Allium cepa L. J. Plant Nutr. 45, 153–167.

[B16] Dong X. C. Qian T. F. Chu J. P. Zhang X. Liu Y. J. Dai X. L. . (2022). Late sowing enhances lodging resistance of wheat plant via improving biosynthesis and accumulation of lignin and cellulose. JIA 22, 1351–1365. doi: 10.1016/j.jia.2022.08.02442574925

[B17] Ehret D. L. Frey B. Forge T. Helmer T. Bryla D. R. (2015). Age-related changes in response of highbush blueberry plants to drip irrigation. HortScience 50, 486–490. doi: 10.21273/hortsci.50.3.486

[B18] Ehret D. L. Frey B. Forge T. Helmer T. Bryla D. R. Zebarth B. J. (2014). Effects of nitrogen rate and application method on early production and fruit quality in high bush blueberry. Can. J. Plant Sci. 94, 1165–1179. doi: 10.4141/cjps-2013-401

[B19] El-Mashad A. A. A. Mohamed H. I. (2012). Brassinolide alleviates salt stress and increases antioxidant activity of cowpea plants (*Vigna sinensis*). Protoplasma 249, 625–635. doi: 10.1007/s00709-011-0300-7 21732069

[B20] Fariduddin Q. Khanam S. Hasan S. A. Ali B. Hayat S. Ahmad A. (2009). Effect of 28-homobrassinolide on the water stress-induced changes in photosynthesis and antioxidant system of *Brassica juncea* L. Acta Physiol. Plant 31, 889–897. doi: 10.1007/s11738-009-0302-730311153

[B22] Feng Y. Y. Shi H. B. Jia Y. H. Li R. P. Miao Q. F. Jia Q. (2023b). Multi-objective optimization water-nitrogen coupling zones of maize under mulched drip irrigation: A case study of West Liaohe Plain, China. Agronomy 13, 486. doi: 10.3390/agronomy1302048630654563

[B21] Feng S. W. Shi C. C. Wang P. Y. Ding W. H. Hu T. Z. Ru Z. G. (2023a). Improving stem lodging resistance, yield, and water efficiency of wheat by adjusting supplemental irrigation frequency. Agronomy 13, 2208. doi: 10.3390/agronomy1309220830654563

[B24] Godfray H. C. J. Beddington J. R. Crute I. R. Haddad L. Lawrence D. Muir J. F. . (2010). Food security: The challenge of feeding 9 billion people. Science 327, 812–818. doi: 10.1126/science.1185383 20110467

[B25] Guo J. Fan J. Xiang Y. Zhang F. Yan S. Zhang X. . (2022). Coupling effects of irrigation amount and nitrogen fertilizer type on grain yield, water productivity and nitrogen use efficiency of drip-irrigated maize. Agric. Water Manage. 261, 107389. doi: 10.1016/j.agwat.2023.10856642574925

[B26] Hao B. Z. Xue Q. W. Marek T. H. Jessup K. E. Hou X. B. Xu W. W. . (2015). Soil water extraction, water use and grain yield by water tolerant maize on the Texas High Plains. Agric. Water Manage. 155, 11–20. doi: 10.1016/j.agwat.2015.03.00742574925

[B27] Hernandez M. Echarte L. Della Maggiora A. Cambareri M. Barbieri P. Cerrudo D. (2015). Maize water use efficiency and evapotranspiration response to N supply under contrasting soil water availability. Field Crops Res. 178, 8–15.

[B28] Jabborova D. Madan A. Perveen K. Khan F. Castañón N. F. Banerjee A. . (2025). Combined application of biochar and arbuscular mycorrhizae enhances growth and soil functionality in common bean under water stress. J. Sustain. Agric. Environ. doi: 10.1002/sae2.7011742587375

[B29] Janeczko A. Gruszka D. Pociecha E. Dziurka M. Filek M. Jurczyk B. . (2016). Physiological and biochemical characterisation of watered and water-stressed barley mutants in the HvDWARF gene encoding C6-oxidase involved in brassinosteroid biosynthesis. Plant Physiol. Biochem. 99, 126–141. doi: 10.1016/j.plaphy.2015.12.003 26752435

[B30] Jayasinghege C. P. A. Bineng C. Messiga A. J. (2024). Effects of long-term nitrogen fertilization and application methods on fruit yield, plant nutrition, and soil chemical properties in highbush blueberries. Horticulturae 10, 1205. doi: 10.3390/horticulturae1011120530654563

[B32] Kuai J. Yang Y. Sun Y. Zhou G. Zuo Q. Wu J. . (2015). Paclobutrazol increases canola seed yield by enhancing lodging and pod shatter resistance in Brassica napus L. Field Crop Res. 180, 10–20. doi: 10.1016/j.fcr.2015.05.00442574925

[B33] Leskovar D. Othman Y. A. (2018). Organic and conventional farming differentially influenced soil respiration, physiology, growth and head quality of artichoke cultivars. J. Soil Sci. Plant Nutt. 18, 865–880. doi: 10.4067/s0718-95162018005002502

[B34] Li J. Yang Q. Shi Z. Zang Z. Liu X. (2021). Effects of deficit irrigation and organic fertilizer on yield, saponin and disease incidence in Panax notoginseng under shaded conditions. Agric. Water Manage. 256, 107056. doi: 10.1016/j.agwat.2021.10705642574925

[B35] Li T. Zhang Y. Bei S. Li X. Reinsch S. Zhang H. . (2020). Contrasting impacts of manure and inorganic fertilizer applications for nine years on soil organic carbon and its labile fractions in bulk soil and soil aggregates. Catena 194, 104739. doi: 10.1016/j.catena.2020.10473942574925

[B37] Liu J. Shu A. Song W. Shi W. Li M. Zhang W. . (2021). Long-term organic fertilizer substitution increases rice yield by improving soil properties and regulating soil bacteria. Geoderma 404, 115287. doi: 10.1016/j.geoderma.2021.11528742574925

[B38] Luo Y. L. Ni J. Pang D. W. Jin M. Chen J. Kong X. . (2019). Regulation of lignin composition by nitrogen rate and density and its relationship with stem mechanical strength of wheat. Field Crop Res. 241, 107572. doi: 10.1016/j.fcr.2019.10757242574925

[B39] Lv F. L. Song J. S. Giltrap D. Feng Y. T. Yang X. Y. Zhang S. L. (2020). Crop yield and N2O emission affected by long-term organic manure substitution fertilizer under winter wheat-summer maize cropping system. Sci. Total Environ. 225, 86–92. doi: 10.1016/j.scitotenv.2020.139321 32438150

[B40] Ma S. C. Wang T. C. Guan X. K. Zhang X. (2018). Effect of sowing time and seeding rate on yield components and water use efficiency of winter wheat by regulating the growth redundancy and physiological traits of root and shoot. Field Crop Res. 221, 166–174. doi: 10.1016/j.fcr.2018.02.02842574925

[B41] Messiga A. J. Dyck K. Ronda K. van Baar K. Haak D. Yu S. . (2020). Nutrients leaching in response to long-term fertigation and broadcast nitrogen in blueberry production. Plants 9, 1530. doi: 10.3390/plants9111530 33182648 PMC7696231

[B42] Messiga A. J. Haak D. Dorais M. (2018). Blueberry yield and soil properties response to long-term fertigation and broadcast nitrogen. Sci. Hortic. 230, 92–101. doi: 10.1016/j.scienta.2017.11.019 33182648 PMC7696231

[B43] Messiga A. J. Nyamaizi S. Yu S. Dorais M. (2021). Blueberry yield and soil mineral nitrogen response to nitrogen fertilizer and nitrification inhibitors under drip-fertigation systems. Agronomy 11, 2144. doi: 10.3390/agronomy1111214430654563

[B45] Naservafaei S. Sohrabi Y. Moradi P. Mac Sweeney E. Mastinu A. (2021). Biological response of Lallemantia iberica to brassinolide treatment under different watering conditions. Plants 10, 496. doi: 10.3390/plants10030496 33807761 PMC8000778

[B46] Ning L. Xu X. P. Zhang Y. T. Zhao S. C. Qiu S. J. Ding W. C. . (2022). Effects of chicken manure substitution for mineral nitrogen fertilizer on crop yield and soil fertility in a reduced nitrogen input regime of North-Central China. Front. Plant Sci. 13, 1050179. doi: 10.3389/fpls.2022.1050179 36589091 PMC9798097

[B47] Okuno A. Hirano K. Asano K. Takase W. Masuda R. Morinaka Y. . (2014). New approach to increasing rice lodging resistance and biomass yield through the use of high gibberellin producing varieties. PloS One 9, 1–12. doi: 10.1371/journal.pone.0086870 24586255 PMC3929325

[B48] Paltineanu C. Coman M. Nicolae S. (2018). Root system distribution of high bush blueberry crops of various ages in medium-textured soils. Erwerbs-Obstbau 60, 187–193. doi: 10.1007/s10341-017-0357-330311153

[B49] Peng D. Chen X. Yin Y. Lu K. Yang W. Tang Y. . (2014). Lodging resistance of winter wheat (Triticum aestivum L.): Lignin accumulation and its related enzymes activities due to the application of paclobutrazol or gibberellin acid. Field Crop Res. 157, 1–8. doi: 10.1016/j.fcr.2013.11.01542574925

[B50] Petridis A. van der Kaay J. Chrysanthou E. McCallum S. Graham J. Hancock R. D. (2018). Photosynthetic limitation as a factor influencing yield in highbush blueberries (*Vaccinium corymbosum*) grown in a northern European environment. J. Exp. Bot. 69, 3069–3080. doi: 10.1093/jxb/ery118 29590429 PMC5972668

[B51] Poorter H. Niinemets U. Poorter L. Wright I. J. Villar R. (2009). Causes and consequences of variation in leaf mass per area (LMA): a meta-analysis (vol 182, pg 565, 2009). New Phytol. 183, 1222–1222. doi: 10.1111/j.1469-8137.2009.02830.x 19434804

[B52] Quemada M. Gabriel J. L. (2016). Approaches for increasing nitrogen and water use efficiency simultaneously. Glob. Food. Secur. 9, 29–35. doi: 10.1016/j.gfs.2016.05.00442574925

[B53] Sekhon R. S. Joyner C. N. Ackerman A. J. Mcmahan C. S. Cook D. D. Robertson D. J. (2020). Stalk bending strength is strongly associated with maize stalk lodging incidence across multiple environments. Field Crops Res. 249, 107737. doi: 10.1016/j.fcr.2020.10773742574925

[B54] Sposaro M. M. Chimenti C. A. Hall A. J. (2008). Root lodging in sunflower. Variations in anchorage strength across genotypes, soil types, crop population densities and crop developmental stages. Field Crop Res. 106, 179–186. doi: 10.1016/j.fcr.2007.12.00142574925

[B55] Strik B. C. (2016). A review of optimal systems for organic production of blueberry and blackberry for fresh and processed markets in the northwestern United States. Sci. Hortic. 20, 103–892. doi: 10.1016/j.scienta.2015.11.04442574925

[B7] Strik B. C. Vance A . (2017). Organic production systems in northern highbush blueberry: I. Impact of planting method, cultivar, fertilizer, and mulch on yield and fruit quality from planting through maturity. Hortscience 52, 1201–1213. doi: 10.21273/hortsci12179-17

[B56] Tian J. Wang C. Xia J. Wu L. Xu G. Wu W. . (2019). Teosinte ligule allele narrows plant architecture and enhances high-density maize yields. Science 365, 658–664. doi: 10.1126/science.aax5482 31416957

[B57] Van Soest P. J. Robertson J. B. Lewis B. A. (1991). Symposium: carbohydrate methodology, metabolism, and nutritional implications in dairy cattle. J. Dairy Sci. 74, 3583–3597. 10.3168/jds.S0022-0302(91)78551-2 1660498

[B58] Wang C. Hu D. Liu X. She H. Ruan R. Yang H. . (2015). Effects of uniconazole on the lignin metabolism and lodging resistance of culm in common buckwheat (Fagopyrum esculentum M.). Field Crop Res. 180, 46–53. doi: 10.1016/j.fcr.2015.05.00942574925

[B61] Wang L. Liu X. L. Liu X. J. Bao X. Y. Zhang X. C. Yin B. Z. . (2024). Effects of spring limited irrigation on grain yield and root characteristics of winter wheat in groundwater-over exploitation areas in the North China Plain. Agr. Water Manage. 294, 108729. doi: 10.1016/j.agwat.2024.10872942574925

[B60] Wang H. Wu L. Cheng M. Fan J. Zhang F. Zou Y. . (2018). Coupling effects of water and fertilizer on yield, water and fertilizer use efficiency of drip-fertigated cotton in northern Xinjiang, China. Field Crops Res. 219, 169–179. doi: 10.1016/j.fcr.2018.02.00242574925

[B59] Wang F. Xie R. Ming B. Wang K. Hou P. Chen J. . (2021). Dry matter accumulation after silking and kernel weight are the key factors for increasing maize yield and water use efficiency. Agric. Water Manage. 254, 106938. doi: 10.1016/j.agwat.2021.10693842574925

[B63] Wei T. Dong Z. Zhang C. Ali S. Chen X. Han Q. . (2018). Effects of rainwater harvesting planting combined with deficiency irrigation on soil water use efficiency and winter wheat (*Triticum aestivum* L.) yield in a semiarid area. Field Crops Res. 218, 231–242. doi: 10.1016/j.fcr.2017.12.01942574925

[B64] Weng F. Zhang W. Wu X. Xu X. Ding Y. Li G. . (2017). Impact of low-temperature, overcast and rainy weather during the reproductive growth stage on lodging resistance of rice. Sci. Rep. 7, 1–9. doi: 10.1038/srep46596 28422161 PMC5395946

[B65] Wu Y. Si W. Yan S. Wu L. Zhao W. Zhang J. . (2023). Water consumption, soil nitrate-nitrogen residue and fruit yield of drip-irrigated greenhouse tomato under various irrigation levels and fertilization practices. Agric. Water Manage. 277, 108092. doi: 10.1016/j.agwat.2022.10809242574925

[B67] Xia G. M. Wang Y. J. Hu J. Q. Wang S. J. Zhang Y. Wu Q. . (2021). Effects of supplemental irrigation on water and nitrogen use, yield, and kernel quality of peanut under nitrogen-supplied conditions. Agric. Water Manage. 243, 106518. doi: 10.1016/j.agwat.2020.10651842574925

[B68] Xu C. D. Tian J. J. Wang G. X. Nie J. K. Zhang H. Y. (2019). Dynamic simulation of soil salt transport in arid irrigation areas under the hydrus-2D-based rotation irrigation mode. Water Resour. Manage. 33, 3499–3512. doi: 10.1007/s11269-019-02312-w30311153

[B70] Yang Y. Luo Y. F. Wu C. L. Zheng H. Z. Zhang L. Cui Y. L. . (2019). Evaluation of six equations for daily reference evapotranspiration estimating using public weather forecast message for different climate regions across China. Agric. Water Manage. 222, 386–399. doi: 10.1016/j.agwat.2019.06.01442574925

[B81] Yang J. C. Zhang J. H. Wang Z. Q. Zhu Q. S. Liu J. J. (2002). Abscisic acid and cytokinins in the root exudates and leaves and their relationship to senescence and remobilization of carbon reserves in rice subjected to water stress during grain filling. Planta 215, 645–652. doi: 10.1007/s00425-002-0789-2 12172848

[B71] Yao C. S. Li J. P. Zhang Z. Liu Y. Wang Z. M. Sun Z. C. . (2023). Improving wheat yield, quality and resource utilization efficiency through nitrogen management based on micro-sprinkler irrigation. Agric. Water Manage. 282, 108277. doi: 10.1016/j.agwat.2023.10827742574925

[B72] Yu N. N. Ren B. Z. Zhao B. Liu P. Zhang J. W. (2022). Optimized agronomic management practices narrow the yield gap of summer maize through regulating canopy light interception and nitrogen distribution. Eur. J. Agron. 137, 126520. doi: 10.1016/j.eja.2022.12652042574925

[B73] Zhai L. C. Zheng M. J. Zhang L. H. Chen J. Zhang J. T. Jia X. L. (2023). Short-term coapplication of organic and chemical fertilizer benefits topsoil properties and maize productivity in a medium-productivity meadow-cinnamon soil. Agronomy 13, 944. doi: 10.3390/agronomy1303094430654563

[B76] Zhang Y. Ma Q. Liu D. Sun L. Ren X. Ali S. . (2018). Effects of different fertilizer strategies on soil water utilization and maize yield in the ridge and furrow rainfall harvesting system in semiarid regions of China. Agric. Water Manage. 208, 414–421. doi: 10.1016/j.agwat.2018.06.03242574925

[B74] Zhang H. Shi Y. Dong Y. Lapen D. R. Liu J. Chen W. (2022). Subsoiling and conversion to conservation tillage enriched nitrogen cycling bacterial communities in sandy soils under long-term maize monoculture. Soil Tillage Res. 215, 105197. doi: 10.1016/j.still.2021.10519742574925

[B75] Zhang S. Xue L. H. Liu J. Jia P. P. Feng Y. Xu Y. . (2024). Onethird substitution of nitrogen with cow manure or biochar greatly reduced N2O emission and carbon footprint in saline-alkali soils. Field Crop Res. 316, 109517. doi: 10.1016/j.fcr.2024.10951742574925

[B77] Zheng M. Chen J. Shi Y. Li Y. Yin Y. Yang D. . (2017). Manipulation of lignin metabolism by plant densities and its relationship with lodging resistance in wheat. Sci. Rep. 7, 41805. doi: 10.1038/srep41805 28150816 PMC5288770

[B78] Zhu M. L. An H. Wu W. (2017). Open field blueberry water and fertilizer management technology. Soil Fertilizer Water Manage., 23–24.

[B80] Zuo W. Q. Wu B. J. Wang Y. X. Xu S. Z. Chen M. Z. Liang F. B. . (2024). Optimal row spacing configuration to improve cotton yield or quality is regulated by plant density and irrigation rate. Field Crops Res. 305, 109187. doi: 10.1016/j.fcr.2023.10918742574925

